# Challenges for Iranian Women in Daily Urban Safety

**DOI:** 10.3389/fsoc.2022.790905

**Published:** 2022-05-09

**Authors:** Fatemeh Hamedanian, Sirwan Ghadermazi

**Affiliations:** ^1^Department of Education, Linnaeus University, Växjö, Sweden; ^2^Independent Researcher, Växjö, Sweden

**Keywords:** public urban spaces, Tehran, women, sense of unsafety, macro and micro level consequences

## Abstract

Ensuring the safety of women as a vulnerable group in urban areas is a fundamental issue and of utmost importance to issues such as violence, crime, victimization, and depression. The purpose of this study is to investigate, through qualitative analysis, the contexts, causes, and consequences of women's feelings of unsafety in urban environments. The research field of the study is the public spaces of Tehran. The subjects and their spatial and interactive dimensions were explored through in-depth individual interviews, direct observation, and participant observation, and data from this study were analyzed using grounded theory. The results show that women's feeling of not being safe in the urban space of Tehran, the capital of Iran, is the result of some influential structural factors such as “socioeconomic challenges” and “dysfunctional socialization” and some contextual factors such as “crowded places” and “showing off.” The women in the study also believe that their feelings of unsafety are reinforced by certain reasons evident in the behavior, language, and gestures of men. The feeling of unsafety among women has consequences at the micro and macro levels. Because of this feeling, women take “preventive measures” at the micro-level and at the macro-level, such feelings of unsafety lead to the spread and reinforcement of the “decay of social trust.”

## Introduction

The experience of urban spaces is a phenomenon concomitant with modernity and has grown more in parallel with the expansion of the modernization process. One of the main aspects of the growth of this phenomenon has been the movement for the de-genderization of places, objects, and speeches in the city because modern urbanization in practice allows the formation of lived experiences for men and women alike. However, some scholars still speak of the sexuality and masculinity of urban spaces (De Simone, 2020). According to these researchers, the shape and appearance of the city is essentially a reproduction of masculine order and gender inequality, and therefore urban spaces are gendered. The most obvious example of this is the chronic fear of women of violence and abuse, which has limited their access to public spaces and the possibility of their mobility in these spaces (Spain, 1993; van den Heuvel, 2019). Thus, for women, experiencing urban spaces is inextricably linked to the experience of feeling unsafe. Women are at greater risk of unsafety due to gender discrimination in society. Several researchers have emphasized that women are more likely than men to feel socially unsafe (Menéndez-Espina et al., 2020), even though women are at significant risk of violence (including rape and domestic violence) in the private sphere. But most women feel more unsafe in public spaces, especially outside the home (Condon et al., 2007).

The dominance of male signs, images, and language over urban space (i.e., interactions, rules, institutions, and procedures) is a source of threat and anxiety for women. The “sexualized nature of urban space” is evident in the function of these spaces to limit women's mobility, a function that generates horror and anxiety in objective behaviors and fear and pessimism in women's minds (Beebeejaun, 2017). The marginalization of women in society and the fact that they do not have access to facilities and opportunities compared to men increase their sense of unsafety in society. It seems that the threat to women's sense of social security is influenced by the structural inequalities and various discriminations in society. Since there are all kinds of cultural and structural inequalities in society, we can try to identify the types of threats that endanger women's sense of social security. According to Koskela (1999), feelings of unsafety and fear shape women's sexual identity by promoting the notion that women must conform to the constraints of societal norms in order to protect their individual safety. Similarly, traditional male sexual identities can reinforce women's fears in public spaces by conveying notions of women's dependence and vulnerability and the dangers of women's unprotected presence in public spaces.

According to some researchers, the discussion of women's experiences in urban spaces today is a debate that is justified and understandable because of the similar conditions and social and cultural proximity in which women often find themselves (Rezazadeh and Mohammadi, 2013; Bhargava and Chilana, 2020; Kern, 2020). Women experience their entire personal and social lives differently. Their lives contain common components and experiences: Inferiority to men, the feeling of not belonging to the male world, fear of the male world, the impossibility of expressing themselves, fear of aggression and violence, being exposed to the gaze of men, and the like. So, the feeling of female unsafety in urban space is not a personal matter. Feelings of unsafety are part of women's everyday life. Everyday life, which according to de Certeau is the result of a combination of actions and whose opaque and pragmatic features resist the application of rational categories (de Certeau, 2011), is the realm of the exercise of power and the emergence of resistance.

Women's sense of unsafety in urban gendered spaces stems from their struggle to be different, to empower their femininity, and to put their stamp on the established and meaningful signs of these spaces, so that women's resistance helps them reject masculine customs and signs. It dissipates the energy of domination and disrupts their system of representation. In everyday life, women associate urban space with two things: Freedom and danger. The most common dangers are fear of rape and sexual assault and other behaviors related to women's loneliness. The “geography of women's fear” provides us with a map of gendered spaces reproduced through spatial functions, a mental and practical geography that causes women to experience urban space in particular ways (Pain, 1997). The transformation of space into a “place of danger” occurs when the interaction of actors is disrupted. In today's urban modernity, women's fear of violent crime is inextricably linked to gendered space. That is, women's fear of public spaces and their precautions in dealing with them stem from the masculinity of spaces (Koskela, 1999). Moreover, women's anxiety is a reflection of gendered power structures in society and also a sign of the power relations in which women are placed (Pain, 1997; Wesely and Gaarder, 2004).

In recent decades, women in Iran have gained prominence in public life. The increasing number of female students and graduates has led to an increase in their demand for work and employment. As women's employment outside the home increases, so does their presence in public spaces. The presence of more women in public spaces has made them feel increasingly unsafe. Various studies in Iran have found that women feel more than moderately unsafe in public spaces in Iranian cities (Alikhah and Najibi, 2006; Modiri, 2006; Bemanian et al., 2009; Heidari et al., 2013). Among the various experiences women may have due to their female body, the experience of feeling unsafe and being afraid of being raped and assaulted is one of the most serious and despite the consideration of security measures for citizens, as it has become a common concern of women in various cities not only in Iran but all over the world. Therefore, it seems necessary to describe and develop this experience in Iran, both to recognize a social phenomenon, the feeling of female unsafety (as part of daily life in our society), and to help women from all walks of life to better understand their situation and not consider their problem as a personal matter. This study aims to achieve the above goal.

## Women's Lived Experience

Although they are the same sex, women of different classes and races have different experiences with their female bodies, and this “exteriority” and “alienation” are also found in men's experiences with their bodies. Thus, although the lived experiences of each gender in the world of life remain inexperienced to the other gender, this particularity does not make the female experience of the female body and its bodily processes universal and generalized. Nevertheless, feminist researchers working within the framework of empirical theories and standpoint theory emphasize the female experience as a keyword, analyzing women's experiences and the material, social, economic, and gendered conditions that produce those experiences (Kruks, 1995; Summers-Bremner, 2000). According to O'Shaughnessy and Krogman (2012), some feminist scholars point to a balance between public interest and visible differences, and such a view allows advocating feminist scholars to use qualitative methods to combat the diversity of women's experiences in their research. In their view, it is more realistic to expect a variety of experiences that overlap with or resemble other women's experiences. In other words, we are less likely to find a common core of similar experiences that are safe from economic conditions, cultural domination, and so on, but rather a set of similarities in a continuum of similarities, so that we may find, for example, significant differences between the experiences of an upper-class, educated woman and an illiterate woman from a slum. Although we acknowledge the significant differences in female thought, we can, in fact, speak of a typological ideal that we call the “women's lived experience” and which, although based on a spectrum of differences, has logical similarities and coherence. This leads us to summarize it under a general heading. Based on this conceptualization of women's lived experiences, the aim of this article is to examine the coherence and similarities of different women's experiences in the public space of the metropolis of Tehran and to draw out their experiences of safety from the web of daily life.

## Theoretical Approach

This study is conducted within a theoretical framework that emerged after the publication of the works of Walter Benjamin, Michel de Certeau, and Henri Lefebvre on modernity and urban space. Benjamin paved the way for twentieth-century critical theory with his discussion of walking in urban areas. He established the link between the walker and the urban landscape of modernity by emphasizing the walker's place in the mass culture of commerce and his inseparable connection to mass biography and mid-century literature. According to Benjamin, modern Paris produced a particular kind of walker whose understanding is linked to that of the passerby. The “flaneur” is a wanderer who enjoys walking through the city without haste. Leisure without a destination is the flâneur's strongest desire. A walk through the city is the best reward that can be given to a flâneur. According to Benjamin, one can see a kind of drunkenness in a person who walks the streets without a goal and for a long time ... Walking creates a great moment with each step (Lauster, 2007). The streets and corridors of the city are the home and refuge of a wanderer who, according to Benjamin, walks on the asphalt (Birkerts, 1982). He is an observer and a vagabond who, like a wanderer, converses with urban space, crowds, and shop windows; the wanderer enjoys walking on the asphalt of the streets. He has chosen to live on the street and wander through the city, joining the crowds, moving around the consumer spaces and staring at the merchandise. He looks at a large number of people, their clothes, their appearance and their faces, and uses facial recognition to distinguish between different classes of people (Paetzold, 2016).

In his work “The Practice of Everyday Life,” Michel de Certeau attempts to develop a theory of production and consumption in everyday life. Everyday life is the realm of power and the formation of resistance. Resistance in everyday life always stems from the differences and otherness of bodies, traditions, and images (de Certeau, 2011). For de Certeau, everyday life has two characteristics: first, that it is repetitive, and second, that it is unconscious. In this context, he introduces two concepts: strategy and tactics. Strategy is the tool of institutions and power structures, and technique is used to find space in the environment defined by strategy. The creation of space is the result of an unconscious attempt to resist formal institutions, discourse, and culture (Schirato and Webb, 1999).

For Lefebvre, the city consists of three components: space, everyday life, and the reproduction of capitalist relations. The city is the common space through which relations of production are reproduced in people's everyday experiences (Fuchs, 2019). Social space is what is formed at the heart of the human experience in daily life and in the culture that governs it. Separating social space from physical space, he highlights the role of culture and the human way of life in this concept, and from this point of view analyzes the city as a legal space in different dimensions. The city is no longer just a living space, but a reflection of the community and social relations mapped on the earth (Unwin, 2000). According to Lefebvre, any kind of social space has three dialectical dimensions (Schmid, 2008): (A) The spatial action or received space, which is the same spatial procedure associated with the method of the physical production of space; (B) The representation of a space or imagined space that has become a conceptual space and is related to the space of scientists, engineers, urban planners, technocrats and social engineers. This is the “dominant space in society” that tends to be a system of linguistic symbols and represents the mental space; (C) The representational space or living space in which consumers live and which is understood through non-verbal means.

Based on this theoretical approach to the subject of this study, it can be deduced that the relationship between the physical, objective, social, and psychological dimensions of urban space is very complex for women. The “invisible invasion” is one of the reasons why they are afraid. In fact, they always have the feeling of being attacked (Monqid, 2012). The fear of crime affects the identity of the space, and the space also affects the fear. Fear of crime changes women's movements and attitudes in urban space (Tandogan and SimsekIlhan, 2016). It restricts their movement so that they stay home, especially after dark; it reduces their social, economic, and cultural opportunities; it increases women's dependence on men, and it reinforces stereotypes about women. This excludes them from social and economic activities such as late or shift work or afternoon classes and the like. Women are the most vulnerable social group when it comes to feeling unsafe in urban spaces. Social monitoring of spaces, revitalizing lost and unprotected spaces, and providing a suitable place for these spaces are among the measures needed to address these deficiencies. Feelings of unsafety include fear of theft, street harassment, fear of criminals and vagrants, assault, violence, and murder. Feeling safe means feeling secure from all forms of aggression, intimidation, and threats to life, property, honor, liberty, employment, housing, and generally all legal and legitimate rights of citizens.

## Literature Review

The background of women's sense of safety in urban environments has been the subject of many Scholar corpus in Iran in the 2000s and before. However, due to a large number of studies, we limited ourselves to studies conducted after 2000. Moreover, to serve the study's aim, we focused only on studies conducted in Tehran urban area.

The results of a study by Nowruzi and Fooladi (2009) on the sense of security of a sample of 384 women aged 15–29 in Tehran showed that only 0.3% of the respondents perceived a high level of social security. Most respondents (52.5%) felt very unsafe in their place of residence, and no respondent felt completely safe in their place of residence. The feeling of social security was moderate in 50.3% of the respondents. This study shows that the variables of feeling safe in the place of residence, economic and social status, social order, and religious affiliation are considered factors that influence the overall feeling of social security.

A study conducted by AmirKafi (2010) on the sense of security of people over 18 living in 22 districts of Tehran concluded that the sense of security in the home was high and very high for 47.2% of respondents and very low for 21%. 31.7% rated their sense of security as moderate. In addition, 9.7% of respondents indicated that their sense of security in Tehran was high and very high. In contrast, 61% of respondents said their sense of security was low and very low and 29.2% said it was moderate. The comparison of the mean scores shows that the feeling of security in the urban community is lower than in the residential areas. In other words, respondents feel more unsafe and afraid outside their residential area.

Afshar (2006) study of women's social security in the 22 districts of Tehran showed that human security and identity security were moderate among women in Tehran, and the sense of social security was below average. Factors such as police performance, law enforcement, social resistance, social support, and social norms influenced feelings of social security. Regression analysis also showed that social security changes when variables such as social support, social norms, and lifestyle change.

Bemanian et al. (2009) examined the factors affecting the promotion of women's safety in the urban environment (around Tehran City Park) and concluded that the average safety in the study area was moderate. The highest security level applies to residential areas or the city park. The lowest security level applies to work areas and production environments. The results show that there is a direct relationship between the perceived safety of space and the traffic volume and use of that space. In addition, to measure indicators of the physical social dimension, the issue of familiarity with the environment and reputation of the space and in the social dimension, the index of social justice of the space and the importance of the variable of gender dominance for the psychological safety of women in this index was discussed.

Safiri (2008) studied the role of neighborhood nongovernmental organizations in providing social security (using Tehran city as an example). The results of this survey on the sense of security in two Tehran neighborhoods^1^ show that most respondents rated the level of financial and personal security as moderate and low. That is, 63% of respondents rated financial security as moderate and low and 37% as high or very high, although life security was rated higher than financial security. However, 43% considered it moderate and low. Most respondents rated the feeling of safety in the neighborhood for family members as low to moderate, with lower scores for children under 10 and higher scores for siblings. This is because the issue of safety, especially for family members in need of care, is a concern for families and the neighborhood is perceived as unsafe. It is said that 26% of children under the age of 10 have a low or very low level of safety and 20% have a moderate level. Even among children aged 10–20 who can take care of themselves to some extent, 27% rate this sense of security as low and very low and 50% as moderate, and among the others the sense of security is somewhat higher.

In the study by Sarukhani and Navidnia (2006) on the social security and place of residence of families in Tehran, none of the families living in the north and south of Tehran overestimated the level of life security at a high level; 27% of families living in the north rated life security as moderate, 65% as low, and 8% as non-existent, while 21% of households in the southern region rated life security as moderate, 67% as low, and 11% as non-existent. Families living in the north have less financial security than those in the south. Of families living in the southern region, 70% rated financial security as moderate, while 28% of families in the northern region rated financial security as moderate. Moral security is almost identical in the two regions, with the difference that 5% of families in the southern region rate moral security as high, while in the northern region many options were not chosen by families. It can be said that the residential area of Tehran families affects four types of security: life, financial, professional, and emotional.

Alikhah and Najibi (2006), after studying women and fear of crime in urban areas in 2006, concluded that overall, about 48% of women in urban areas have a high fear of the threat of crime, 33% have a medium fear, and 19% have a low fear. In other words, about half of the women did not feel safe when they were out and about in the city. The women surveyed felt safer in their neighborhoods than in other areas. Respondents' reported experiences with unsafety showed that 90% of respondents were stopped and harassed while driving, 80% were body touched and pushed, 70% had bags and cell phones stolen, and all women were taunted and teased. Forty-six percent of respondents feel safe going to the mountains, 36% to the movies and the park, 24% to the gym, and 33% to the market.

## Methodology

The purpose of this study is to qualitatively examine the causes, contexts, and consequences of women's feelings of unsafety in Tehran, the capital of Iran. In order to investigate this topic in-depth, the grounded theory method was used. This method, whose results cannot be obtained through statistical operations or other counting methods, is essentially about interpretation. This interpretation is used to discover concepts and relationships in the raw data and organize them in the form of a theoretical explanatory plan. . Data are usually obtained from interviews and observations, but may also include documents, videos, and videotapes (Corbin and Strauss, 1990). The main reason why the researcher chose this method is because of the nature of the research question.

This study was conducted in Tehran in 2021. A random sample was drawn and the two criteria “reaching the theoretical saturation point” and “diversity” were used to determine the sample size. A total of 50 respondents were interviewed (Table 1). The interviewees or key informants were those who had the most power in clarifying the research question. In addition, care was taken in the selection of individuals to ensure that they were as diverse as possible with respect to some dimensions and contextual indicators. In-depth individual interviews were conducted to collect data. After the interviews were recorded and translated, their content is applied to these sample sentences in the form of four stages of the coding process (conceptualization, concept reduction, categorization, and relevance). Finally, based on the conceptual arrangement that emerged from this process, an explanation of the women's experiences of feeling unsafe in urban spaces was provided (Table 2). It should be noted that the process of coding and data analysis was conducted manually, which seemed more appropriate given the interpretive and in-depth nature of the research. In addition, qualitative principles and criteria were used to evaluate accreditation. The most important evaluation criterion here is “reliability,” which has four dimensions: “dependability,” “reliability,” “verifiability,” and “transferability” (Lichtman, 2014). The use of the strategy of “triangulation” was evaluated. Triangulation is the consensus on a particular outcome using a combination of different methods, theories, and data. In general, an attempt was made to explore the reasons, contexts, and consequences of “feeling unsafe in urban spaces” using in-depth personal interviews. Semi-structured interviews are most appropriate for getting stakeholders to talk about these experiences. On the other hand, the researchers aimed to ensure the greatest possible diversity and variety in the study sample and tried to select interviewees from different age groups and strata to enrich the content of the study.

**Table 1:** Background of interviewees.

**Contextual variables**		**Frequency**	**Percent**
Age	18–25	9	18%
	26–35	24	48%
	36–45	12	24%
	46–55	5	10%
	56–60	–	–
Education	High school Diploma	28	56%
	College and Bachelor Diploma	18	36%
	Masters and Ph.D.	4	8%
Economical class	Low	16	32%
	Average	30	60%
	High	4	8%
Employment	Employed	15	30%
	Unemployed	35	70%

**Table 2:** Data coding.

**Sub- pivotal categories**	**Pivotal categories**	**Major categories**	**Core theme**
Financial obstacles	Socio-economic challenges	Structural factors	
Social straits			
Men and women messy path of socialization	Dysfunctional socialization		
Men frustration			
Inevitable interactions	Crowded places	Context-related factors	
Body management and behavior	Showing-off		Sense of unsafety
Verbal harassment	Scratchy relationships	Reasons	
Chasing behaviors			
Physical harassment	Stalking behaviors		
Sexual gaze			
Adopt deterrent strategies/silence	Preemptive measures	Consequences	
Inter-personal and organizational distrust	Deterioration of social trust		
Reproduction of violence and inequality	Discrimination and street violence		

## Results

As a result of the analysis of the interviews and field observations, the researchers found that feeling safe is a major challenge for women in urban areas of Tehran and is associated with the experience of having a female body in daily life. Women's fear of urban space manifests itself in different ways at different times of the day, and the women interviewed expressed their sense of unsafety by naming specific causes and factors in Tehran's urban space. From the analysis of the concepts in the interviews and the places where they expressed their concrete experiences of feeling unsafe in the city, it was concluded that women feel unsafe when they are in urban spaces due to structural problems in Iranian society and contextual reasons. In all these cases, women are forced to choose options that have consequences at the micro and macro levels. According to the results of the interviews, it can be said that between 76 and 85% of the interviewed women consider the security in the urban area of Tehran to be weak and very weak. In the following, the results of the interviews are discussed in detail.

### Structural and Context-Related Factors

To answer the question, “What are the causes and contexts of harassment and Unsafety for women in Tehran urban spaces?” Women were interviewed as actors in public spaces and as the main experiencers of this phenomenon. Relevant concepts were then extracted from their statements through an open, axial, and selective coding process.

#### Socio-Economic Challenges

According to some researchers, actors' violations of social norms result from structural challenges and a lack of moral order in society. When the needs and desires of individuals and groups in society do not match the available gifts and rewards, such conflict provides a backdrop for the deviance of some members of society (Shannon, 2000). Merton considers anomie as a natural reaction of people to their living conditions. According to him, behaviors such as conformity, retreat, ritualism, innovation, and rebellion are exhibited in response to the conflicts that arise between socially accepted values and the limited opportunities to access those values (Merton, 1938).

The reactions of many women who are harassed in the public show the effects of socio-economic challenges reproduced by men's behavior:

“I think that high unemployment among young men is one of the main reasons because an unemployed man comes out and harasses other women.” (Code 3).

“If the relationship between a girl and a boy becomes freer and it's easier for them to go out together and talk to each other, it will not be like that. I think society has put so many restrictions on young people.” (Code 22).

It can be vividly stated that the women who have been victims of harassment and insecurity in public spaces agree on the structural challenges in society and their impact on the occurrence of such behaviors in the social context and that one of the most important ways to curb this phenomenon is to solve the structural problems in society.

#### Dysfunctional Socialization

Some respondents' statements about the roots of some behaviors point to problems that arise when people are socialized.

“I think it's because of the family and how they raise their sons. Some of these men do not know the status of women and how to treat them.” (Code 43).

“All sorts of things can be said about grass people who have no family. It vividly shows that they did not grow up in a solid family environment.” (Code 8).

“In my opinion, we are more harassed and insecure because women are not supported in society or because people see women in a special way. In such a community, it does not matter if a girl is modest and quiet, men will pursue her because of her body. More than ninety percent of them do this for fun.” (Code 15).

“In society, most people see women from a sexual point of view. If you go out and are alone, they treat you like a second-hand commodity. In a way, they are right because they have grown up in a society where they have internalized that.” (Code 32).

Many women who told us about their experiences pointed out the attitudes that exist and are sometimes promoted among women in families and society. The traditional structure of families does not allow women to express themselves in many areas, and in many cases, they are further limited by the stereotypes that arise from the local context. In this regard, the socialization process takes place in an abrupt and massive context, i.e., social changes and a growing level of anomie in society lead to dysfunctional socialization that arises from the conflict between the law and other aspects of social life to maintain the unequal social order between women and men (Lehmann, 1995) in a society like Iran. Another dimension of the socialization process of women that can be derived from the interviews is the process of failure and negative feedback from society. The reference to anomie, disorder, and its consequences, such as inequality, is the central axis that negatively affects socialization. In this process, women feel constantly frustrated because they feel they have to change and are disadvantaged in their interaction with men. This feeling has a decisive influence on disrupting their communication process and their feeling of unsafety and pessimism toward men as “others.”

“Most of them have sadism. If someone has even an ounce of sense, he will not do that. I am sure that someone who molests his mother and sister will say nothing and put up with it. They do not care about anything, neither their personality nor the personality of others.” (Code 28).

“All women believe that they are people who have mental problems because of their sexual deficiencies and emotional problems that bother women and girls.” (Code 17).

Based on these interpretations, it can be said that the two phenomena of dysfunctional socialization of men and women and communication frustration of men as part of fundamental structural factors in a fortuitous combination provide the reason why women feel increasingly unsafe. In such an atmosphere, the “other” is a heterosexual taboo to be deciphered. All the movements, the language, and the signs of this “other” have complex meanings to which any reaction, especially in unfamiliar urban spaces, is gratifying and produces a kind of victory. In this public space, it is not easy for a woman to accept the culture and social customs and gain socio-cultural capital, but she is constantly afraid of everything that has a different color and the smell of heterogeneity and male company.

#### Crowded Places

Respondents' interpretation of their daily experiences in public spaces sometimes implies that heterosexual contact is inevitable in modern urban spaces. Therefore, despite the existence of fears, precautions, and other obstacles, there is no way out to accept the existence of the “other” and to regulate and control one's own body and mind in the interaction with him. From the women's point of view, some places such as streets with shopping malls, cafes, cabs and buses, stores, and other important areas as contextual factors favor the feeling of unsafety. The presence in these places is a kind of double-edged sword for women: a tool for freedom and communication, while at the same time they feel dangerous.

“There are some (guys) who may really like the girl on the street and want to tell her that because they have no other choice.” (Code 5).

“Suppose a girl wants to get married, where can she meet and talk to a boy! At home she cannot find her partner, she must go out. Outside is very good, but I wish there were not all these fears and anxieties.” (Code 42).

“I am sad because if it were possible for a girl and a boy just to go out and talk to each other, they would never approach people and bother them. A number of restrictions force them (guys) to express themselves in this way.” (Code 17).

#### Showing-Off

Public spaces have always been the realm of physical and symbolic representation of people in the presence of others. Respondents' interpretations of these spaces sometimes suggest that their representation is not only a goal but also a pleasure for them. However, the fun and enjoyment of this action lead them to confront a sense of unsafety. Thus, the two contradictory aspects of the lived experience of presence and action in urban space are inseparable in the lives of women. For example, more than half of the respondents indicated that the type of clothing and makeup they wear has an impact on harassment or unsafety.

“I think the more makeup she wears and the worse her clothes are, and she will arouse the more men out there.” (Code 7).

“Yes, clothing and grooming have an effect. You cannot say that they have no effect. The women who come with an improper dress are indeed wearing it to attract the attention of others (men) so that the boys will see them and take pleasure in their dress.” (Code 14).

“A woman's dress and type have a great influence on how she is treated by men. The tighter the clothing and the more provocatively a woman is dressed, the more she is harassed by men/boys.” (Code 22).

### Reasons

The epistemological premise of grounded theory analysis is based on the fact that what people feel and perceive as reality emerges through their symbolic interaction. Sociologists must therefore analyze the processes of reality construction rather than falsifying the concept of social reality itself. According to Berger and Luckmann (1991), our task is to analyze the processes by which people arrive at what seems natural to them. Accordingly, the current research focuses on examining the experiences of women living in urban public spaces and, in particular, the reasons they give for feeling unsafe in these spaces. Of course, women do not have the same perceptions and interpretations of being in these spaces, and the reasons they give for their constant fear of being physically present in these spaces vary. So, we attempt to extract key concepts from their various interpretations and form a set of pivotal and major categories from these concepts to arrive at a deeper understanding and interpretation of women's feelings of unsafety in these spaces.

#### Scratchy Relationships

The two categories of verbal harassment and chasing behaviors can be grouped under the main category of “scratchy relationships.” These two categories, which are the result of understanding and interpreting the reasons for women's unsafety in urban public spaces, show the decline of dialog, distorted interactions, and other fears between the two sexes in the current situation of Iranian society, “scratchy relationships” are the product of a space where any normal and ordinary communication with the other encounters all kinds of obstacles, misunderstandings, and misconceptions, and inevitably opens the way to distorted and incomplete forms of communication, such as verbal harassment. In this atmosphere, unspoken rules of conversation are violated, intentionally or unintentionally. The discomfort caused by this situation leads to a feeling of unsafety among the women. The concepts contained in the interviewees' narratives clearly show the various forms of this violation of the rules of dialogue and communication. The following are examples of verbal harassment:

- Insulting

“If you do not take them up on their offer, they start snarking and saying, 'You are all such a bitch, and there are a thousand like you.” (Code 3).

- Dating offer

“First they offer sex. It was night, I got in the car (it was not a cab), halfway to my destination the driver said how much do you get for sex? I said I do not understand. He said, Do not be shy, I know women like you, and started cursing, whereupon I got out with a miserable demeanor.” (Code 34).

- Offer his phone number

“We were at the park with my friends. The guy came up to me and said, 'Please get my number and call me right away so we can make a friendship.” (Code 16).

The interviewees' statements show that one of the most common harassments for women in public spaces today is verbal harassment, harassment that is less risky for the perpetrators and, because of its transience and anonymity, encourages harassers to use this type of harassment. And are considered normal due to the multitude of these expressions in public spaces and have no such consequences for the perpetrators.

“Chasing behaviors” is the second category that falls under the main category of “scratchy relationships” cultivated in walking and driving. This common practice indicates a lack of healthy space for everyday communication between the sexes and a solid male desire to break the taboo of heterosexuality (woman as the other). “Chases” are a new urban phenomenon driven mainly by unbridled sexual desire. Women are most afraid of this phenomenon and it shows in their narratives:

Stopping the car

“The car stopped in front of me, and the man said, 'Come and get in.' I thought that was a sexual proposition.” (Code 10).

- Chase by car

“The guy chased me with his car, so I took off, but he kept doing it until he finally showed up at my front door, and after I went inside my house, he disappeared after a while.” (Code 12).

- Chase on foot

“Once when I was shopping with my friend, a guy chased us, I cursed him, but he did not give up. So I took my friend's defensive knife from his pocket and was about to stab him when I realized he was running away.” (Code 48).

#### Stalking Behaviors

The phenomena of the “sexual gaze” and “harassment” take place in an environment in which it is not only difficult to establish a normative relationship between the sexes but also in which the satisfaction of the basic needs and instincts of individuals encounters serious obstacles. This reinforces a variety of alternative responses, such as stalking behaviors. According to Maslow (1954) and Baumeister (1992) when the “ego” is weakened under the pressure of the demands of the “superego” and the noble sides of being human, such as the power of judgment and decision and love, are increasingly suppressed, people resort to instincts. Sexual gaze occurs in the context of interactions in which individuals view each other as sexual objects because of recourse to instincts. In today's consumer society, this is both influenced by the media, the feminization of space and objectivity, and reinforced by the absence of dialogue, communicative action, and the taboo of love and sexual intimacy. In a society where there is an apparent conflict between real life and the world presented in the virtual world, such as satellite channels and the Internet, the sexual gaze becomes increasingly problematic. These conflicts and tensions limit the communication process and increase aggression and violence. Accordingly, “sexual gaze” and “physical harassment” symbolize a form of sexual aggression and violence resulting from various cultural and communicative conflicts. The interviewees' statements serve as data to confirm the aforementioned analysis:

- Staring

“They (men) often stare at women, it's really like that, it's even happened that I have gone shopping with my husband, but many men have stared at me.” (Code 22).

- Touching body

“One afternoon I went to a friend's house with my other friend. I was waiting for her on the street near my house when suddenly a man on a motorcycle approached me and came so close that I was lying against the wall. He put his hand in front of me and molested me until I started to cry.” (Code 33).

- Physical violence

“I had an experience with an attack. It was a winter day, and there was rarely a cab. I decided to walk home. Near my house, a man attacked me from behind and touched me all over my body.” (Code 37).

### Consequences

In this section, we attempt to answer what consequences the sense of unsafety in public space had on women's movements and social life in public space and how they described the consequences they experienced in their personal and collective lives. Based on the interviewees' responses, we tried to extract the main concepts and categories and carry out the coding process in this phase.

#### Preemptive Measures

Examination of the participants' statements show that in many social situations where they feel unsafe and harassed, they try to use strategies and actions to minimize unsafety and harassment. However, of course, respondents acknowledged that using these strategies did not reduce the severity, type, or frequency of the harassment in many cases. This leads us to an essential point behind these answers, which is that harassment is widespread among women, despite differences in appearance, dress, and make-up. Some of these preemptive measures are:

- Adopt a deterrent strategy

“If I am walking lonely on a path and there's a bunch of guys over there, I'd rather turn around and go the other way.” (Code 28).

- Silence

“I prefer to remain silent because of my reputation. Women like me do not dare to tell their family because I am afraid they will soon condemn me.” (Code 41).

#### Deterioration of Social Trust

One of the negative consequences of unsafety is the loss of mutual trust between strangers and anonymous people. As a result, the emergence of pessimism and refusal to communicate with the “other” in social interactions is widespread among women. This means that they do not trust other people around, nor the authorities and institutions responsible for security in the public space. It is also an indication of the exhaustion of the social capital of individuals and institutions. Social capital is a type of communication asset in which actors create and mobilize communication networks within and across organizations to access the resources of other social actors. In other words, social capital (Woolcock and Narayan, 2000) enables actors to live more comfortably thanks to the support they receive from others and the opportunities they already have thanks to their relationships with others. But, on the other hand, as social capital declines, we will also see the deterioration of social trust, which, according to the results of this study, can be seen in two dimensions:

- Undermining interpersonal trust

“Many of these troublesome types do not care whether a woman is single or married, but harassment has become like a job to them. These harassments have even led me to be pessimistic about many men and say they are all the same.” (Code 11).

- Distrust of the security institutions

“I noted the license plate number of the troublesome man and informed the police, I followed up the case, but unfortunately without any result with the police.” (Code 19).

#### Reproduction of Violence and Gender Inequality

Gender is socially created and has a decisive influence on people's roles and social identity. Gender differences are not neutral and are one of the fundamental pillars of social stratification. Gender is also an essential factor in creating a diversity of possibilities and opportunities in individuals' lives. The gender division of work creates inequality between men and women regarding power, prestige, and wealth. This gender inequality, which is both the product of symbolic violence and an exacerbation of that violence (Leacock, 1992), is reproduced in different ways in the daily interactions of the two sexes in urban and social gendered spaces. In other words, the feeling of female unsafety is dialectically linked to the reproduction of violence. In other words, this feeling itself is both a symbol of a type of violence and the reason for the continuation and reproduction of violence and gender inequality. The results of the data show that this type of violence and the resulting unequal context manifest in two ways:

- Street violence

“Once, I was harassed by a troublesome man. I was out with two of my friends, got into an argument with him, and called him everything in the book. The guy was stuck like a leech and would not let up. My friend got a knife out of his backpack, but my other friend and I threw rocks at him, after which he ran away.” (Code 29).

- Reproduction of gender discrimination

“In my opinion, harassment and insecurity are increasing for us, and the situation is not improving because women are not supported socially and legally. Due to an unequal structure in which we live as women, men have a particular standpoint of women, and it is only natural that men regard women as the second sex and rank them lower than themselves.” (Code 15).

## Conclusion

Despite its simplicity and triviality, everyday life has certain complexities and features that make it difficult for the people who live in it to understand the details. In most cases, the everyday knowledge of individuals and social groups is limited to their respective social and economic status and living conditions. In addition, socially disadvantaged groups are less willing to talk about their particular situation and the experiences and feelings associated with it. Sometimes certain situations have characteristics that make it difficult or inadmissible to talk about them. The female body and women's feelings about having this body, as well as their experiences with it in daily life, are among these themes. Among the many problems women face because of their female bodies, one of the most important and controversial is the feeling of unsafety and the fear of being raped in the social space of the city. So, in urban spaces with very complex physical, social, and psychological dimensions, women are exposed to invisible invasion and symbolic aggression, which is the reason for their fear. This is a problem that affects not only a particular society but also many countries, and it has been addressed in women's literature and from a sociological point of view. So, the experiences of women and their details can be revealed when the women who have experienced them themselves report such an experience.

The main question of this study was: what are the primary contexts, reasons, and consequences of women's feelings of unsafety? To answer this question, we used grounded theory analysis and techniques such as face-to-face in-depth interviews, direct observation, and participant observation to examine the study site (the public spaces in Tehran) and its spatial and interactive dimensions. Based on targeted sampling, data were collected and analyzed using the coding process.

As can be seen, women's sense of unsafety in the public spaces of Tehran, the capital of Iran and a metropolis, is rooted in a structural-contextual basis that can be divided into two types; (A) Structural factors that play a crucial and direct role in the development of women's sense of unsafety, including “socio-economic challenges” and “dysfunctional socialization.” And (B) the contextual factors that play a role in creating the preliminary background for women's feelings of unsafety. For example, “crowded places” lead to the inevitable interactions between men and women. Women's tendency to “showing-off” to men is also among the factors that increase feelings of unsafety. The women interviewed also believe that their feelings of unsafety are reinforced by specific reasons that are evident in men's actions, speech, and gestures; the main reasons are the “scratchy relationships” of men with women, which take the form of verbal harassment and chasing behaviors due to the obstacles and restrictions in establishing a relationship with women. Moreover, the “stalking behaviors” that result from these scratchy relationships, as well as sexual gaze and physical harassment, are the most prominent examples.

**Figure 1:**
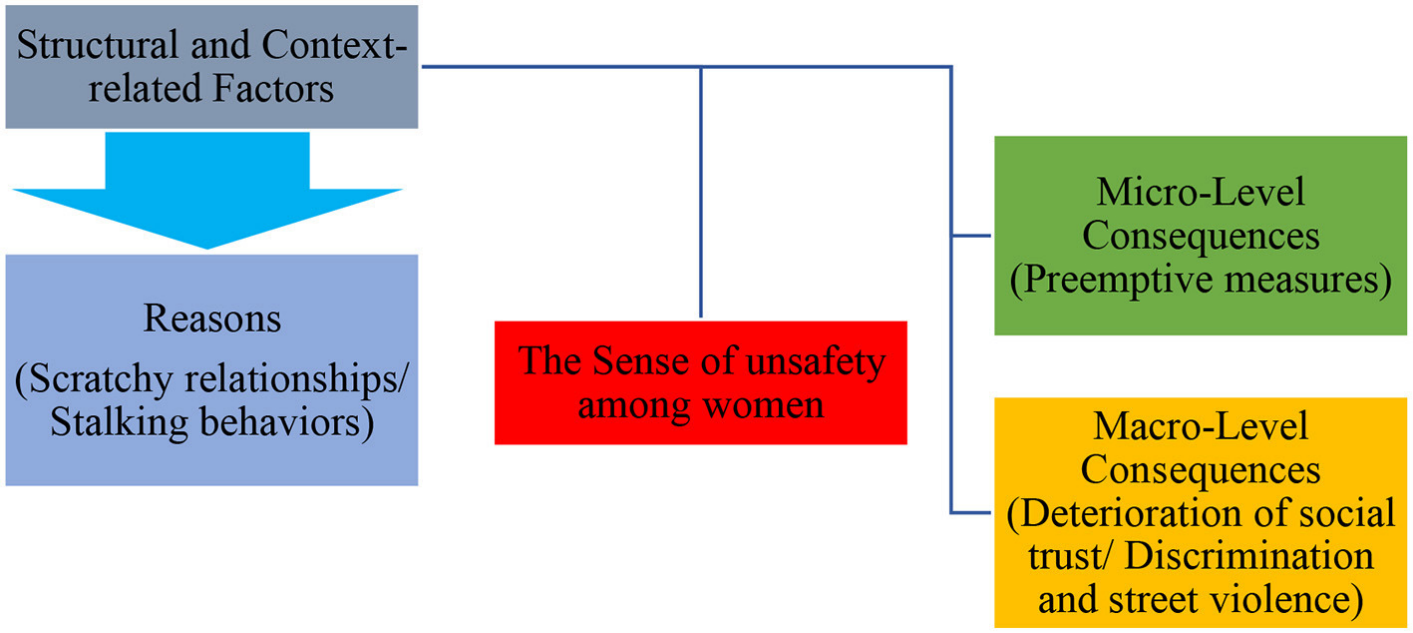
Background model on women's experience of feeling unsafe in public spaces in Tehran.

In addition, women's sense of unsafety has micro-and macro-level implications. Because of this feeling, women resort to “preemptive measures” at the micro-level (their personal lives), meaning they are forced to use a range of deterrent strategies and choose tactics such as silence. However, the consequences of this sense of unsafety at the macro level are also significant, i.e., it leads to the spread and reinforcement of the “deterioration of social trust” both at the “Undermining interpersonal trust” and “distrust of security institutions,” resulting in the reproduction of violence (street violence) and gender inequalities (Figure 1).

There are some noteworthy points in a general conclusion from the women's approach. First, the behavior of all actors (including women) in practice involves a combination of resistance and obedience. Thus, even women who called themselves feminist and openly opposed the norms of the female body sometimes reacted differently. For example, they preferred to leave the night with all its possibilities and pleasures to men, avoiding pleasurable and at the same time dangerous areas such as walking on a secluded street and secretly submitting to male norms. Alternatively, they showed creativity rather than resistance, preferring, for example, to be on the street at night with a group of women. Conversely, traditional women, for example, who tend to obey male norms, may sometimes resist and leave the house, for example, to go to a place (such as a mosque or a party) that is not in sight of their husbands. Moreover, although often described as a hostile and dangerous environment for women, the urban environment can also be seen as a prerequisite for the liberation of women, promoting the femininity of the urban environment with attractive freedoms and numerous opportunities. Last but not least, the constant feeling of unsafety for women in public spaces is an uncomfortable, painful, and humiliating feeling that both confirms and exacerbates the social inequalities that exist in the relationship between men and women. That being said, the issue of rape and the possibility of being abused is so firmly and influentially entrenched in the female consciousness that resistance to it fades in most cases, and women inevitably or secretly submit to men's rules of the game and adhere to some restrictive norms.

## Data Availability Statement

The original contributions presented in the study are included in the article/supplementary material, further inquiries can be directed to the corresponding author/s.

## Ethics Statement

Ethical review and approval was not required for the study on human participants in accordance with the local legislation and institutional requirements. The patients/participants provided their written informed consent to participate in this study. Written informed consent was obtained from the individual(s) for the publication of any potentially identifiable images or data included in this article.

## Author Contributions

All authors listed have made a substantial, direct, and intellectual contribution to the work and approved it for publication.

## Conflict of Interest

The authors declare that the research was conducted in the absence of any commercial or financial relationships that could be construed as a potential conflict of interest.

## Publisher's Note

All claims expressed in this article are solely those of the authors and do not necessarily represent those of their affiliated organizations, or those of the publisher, the editors and the reviewers. Any product that may be evaluated in this article, or claim that may be made by its manufacturer, is not guaranteed or endorsed by the publisher.

## Footnotes

^1^Amiriyeh and Shahrak-e-Jandarmeri.
